# Decreased Colorectal Cancer Incidence and Incidence-Based Mortality in the Screening-Age Population of Ontario

**DOI:** 10.1093/jcag/gwaa035

**Published:** 2020-10-15

**Authors:** Lawrence F Paszat, Rinku Sutradhar, Elyse Corn, Jill Tinmouth, Nancy N Baxter, Linda Rabeneck

**Affiliations:** 1 Institute for Healthcare Policy Management and Evaluation, University of Toronto, Toronto, Ontario, Canada; 2 Dalla Lana School of Public Health, University of Toronto, Toronto, Ontario, Canada; 3 Cancer Research Program, ICES, Toronto, Ontario, Canada; 4 Department of Medicine, University of Toronto, Toronto, Ontario, Canada; 5 Department of Surgery, University of Toronto, Toronto, Ontario, Canada

**Keywords:** Colonoscopy, Colorectal cancer incidence, Fecal occult blood testing, Incidence-based mortality

## Abstract

**Background and Aims:**

We aimed to evaluate trends in Ontario, Canada, 2002 to 2016, in uptake of colorectal evaluative procedures, colorectal cancer (CRC) incidence and incidence-based mortality in the colorectal screening-age population.

**Methods:**

We defined the screening age-eligible population as persons 51 to 74 years of age with ≥1 year eligibility for the Ontario Health Insurance Plan, excluding those with a diagnosis of CRC in the Ontario Cancer Registry (OCR) prior to age 50 or January 1, 2002. We computed annual up-to-date status with colorectal evaluative procedures from billing claims, and CRC incidence from the OCR. In order to compute incidence-based CRC mortality, we included persons with a first diagnosis of CRC between the ages of 51 and 74, diagnosed between January 1, 1992 and December 31, 2001, still alive and <75 years of age on January 1, 2002, based on cause of death from the OCR. Overall, age-stratified and sex-stratified trends were evaluated by Cochran–Armitage trend tests.

**Results:**

Persons up to date with colorectal evaluative procedures increased from 628,214/2,782,061 (22.6%) in 2002 to 2,584,570/4,179,789 (62.2%) in 2016. CRC incidence fell from 129.3/100,000 in 2002 to 94.54/100,000 in 2016, and incidence-based CRC mortality fell from 40.8/100,000 to 24.1/100,000. Decreasing trends in overall and stratified incidence and mortality were all significant, except among persons 51 to 54 years old.

**Conclusions:**

There was continued increase in persons up-to-date with colorectal evaluative procedures, and significant decrease in CRC incidence and incidence-based CRC mortality from 2002 through 2016.

## Background

Statistically significant declines in age-standardized colorectal cancer (CRC) incidence stratified by site and sex have been documented in Canada since 1983 for all combinations of colorectal subsites and sex, with the exception of right-sided colon cancer among males, for whom a statistically significant annual percent change increase in age-standardized incidence occurred between 1983 and 2007. In Ontario, overall age-standardized CRC incidence declined on average 0.8% annually between 1998 and 2007 (0.4% among males and 1.0% among females) and age-standardized CRC mortality declined on average 1.7% annually between 1997 and 2006 (1.7% among males and 1.9% among females) (Canadian Cancer Statistics 2011) (1).

Subsequent to a meta-analysis of trials of colorectal screening using fecal occult blood testing (FOBT) demonstrating significant reduction in CRC mortality in 1998 (2), two Canadian guidelines recommending biennial FOBT were issued in 2001(3) and 2002(4). At the time, there was evidence of substantial use of colorectal evaluative procedures among screening-age persons in Ontario without prior diagnoses of bowel diseases (5–7). In 2008, Ontario established ColonCancerCheck (CCC, a population-based colorectal screening program for persons at average risk for CRC aged 50 to 74 years recommending biennial FOBT, and screening by colonoscopy for those with a first-degree relative affected by CRC) (8).

In this paper, we aim to evaluate trends in colorectal evaluative procedures, CRC incidence and incidence-based mortality in the screening-age population of Ontario, 2002 to 2016.

## METHODS

This work was approved by the Research Ethics Board of Sunnybrook Health Sciences Centre, Toronto, Ontario (REB 396-2017).

### Identification of the Cohort of Colorectal Screening-Eligible Persons

We identified persons 51 to 74 years of age with ≥1 year eligibility for the Ontario Health Insurance Plan (OHIP) during 2002 to 2016 based on OHIP’s Registered Persons Database (RPDB), excluding those with a diagnosis of CRC in the Ontario Cancer Registry (OCR) age <= 50 years or diagnosis prior to 2002. Records of FOBT were identified from the OHIP billing claims file and CCC’s FOBT database. Flexible sigmoidoscopy (FS), CT colonography (CTC, data available since 2011 only) and colonoscopy were identified from the OHIP physician billing claims file. The intended indications are unavailable in the data. CRC was identified from the OCR. All persons were followed until December 31, 2016, their 75th birthday, last date of OHIP eligibility, first diagnosis of CRC or date of death, whichever came first.

### Computation of Up-to-Date Status With Colorectal Evaluative Procedures

For each year, 2002 to 2016, we computed the up-to-date status of each person with colorectal evaluative procedures. Being up-to-date with FOBT was given priority over any other colorectal evaluative procedure performed during the 24 months following the date of the FOBT. If a person had a record of FOBT ≤24 months prior to the end of the year, but no record of colonoscopy within the prior 10 years or any record of CTC or FS within the prior 5 years, the person was classified as up-to-date with FOBT. If not up-to-date with FOBT, but with a record of FS ≤60 months prior to the end of the year, with no colonoscopy within the prior 10 years or CTC within the prior 5 years, the person was classified as up-to-date with FS. If not up-to-date with either FOBT or FS but with a record of CTC ≤60 months prior to the end of the year, with no colonoscopy within the prior 10 years, the person was classified as up-to-date with CTC. If not up-to-date with FOBT, FS or CTC but with a record of colonoscopy ≤10 years prior to the end of the year, the person was classified as up-to-date with colonoscopy. Otherwise, the person was classified as not up-to-date with any colorectal evaluative procedure. For each calendar year, the percent up-to-date (overall and stratified by age and by testing modality as above), and the percent not up-to-date with any test, was computed and plotted. We also computed the percent up-to-date with FOBT among a subpopulation of those who were not up-to-date with colonoscopy.

### Computation of CRC Incidence Among Screening-Eligible Persons

We extracted the date of the first diagnosis of CRC and the diagnosis code (International Classification of Diseases, version 10 (ICD10): C18 [colon], C19 [rectosigmoid] or C20 [rectum]). For each year, the rate of CRC per screening-eligible 100,000 persons was computed, with 95% confidence intervals (CIs), overall, and stratified by age, sex, and by ICD10 code C18 (colon) versus ICD10 codes C19 plus C20 (rectosigmoid plus rectum). We computed Cochran–Armitage trend tests on incidence from 2002 to 2016, overall, stratified by sex and stratified by age groups.

### Computation of Incidence-Based CRC Mortality

To compute incidence-based CRC mortality (9,10) among persons aged 51 to 74 during 2002 to 2016, we identified the underlying population at risk for CRC death in the screening age-eligible age range by including persons diagnosed with CRC in the colorectal screening age range prior to January 1, 2002, still alive on that date, and still in the age range 51 to 74 years, followed until December 31, 2016, their 75th birthday, last date of OHIP eligibility or date of death, whichever came first. Cause of death was identified from the OCR. For each year, incidence-based CRC mortality was computed with 95% CIs, overall, stratified by age and sex. We computed Cochran–Armitage trend tests on incidence-based mortality from 2002 to 2016, overall, stratified by age and stratified by sex. All analyses were performed using SAS version 9.3.

## RESULTS

### The Colorectal Screening-Eligible Persons, 2002 to 2016

Colorectal screening-eligible permanent residents of Ontario increased from 2,782,061 (51.3% female) in 2002 to 4,179,789 (51.2% female) in 2016. Over 90% had been eligible for OHIP ≥10 years on the date of initial inclusion in the study population. Table 1 describes the characteristics of the screening-eligible population for several years surrounding 2008, the year of CCC implementation, as well as the first and final years of the observation period.

**Table 1.** Screening-eligible population and up-to-date status at key calendar years

Year	2002	2005	2006	2007	2008	2009	2016
Permanent residents in the screening-eligible population	2,782,061	3,044,523	3,129,595	3,197,332	3,281,028	3,386,397	4,179,789
Age groups							
51–54	810,730 (29.1%)	865,863 (28.4%)	893,132 (28.5%)	913,766 (28.6%)	939,021 (28.6%)	967,115 (28.6%)	1,108,258 (26.5%)
55–59	637,926 (22.9%)	753,627 (24.8%)	787,132 (25.2%)	789,194 (24.7%)	792,506 (24.2%)	810,703 (23.9%)	1,003,582 (24.0%)
60–64	501,040 (18.0%)	562,457 (18.5%)	578,340 (18.5%)	613,606 (19.2%)	654,246 (19.9%)	686,600 (20.3%)	835,479 (20.0%)
65–69	439,590 (15.8%)	459,674 (15.1%)	468,888 (15.0%)	475,794 (14.9%)	491,370 (15.0%)	510,265 (15.1%)	720,506 (17.2%)
70–74	392,776 (14.1%)	402,903 (13.2%)	402,104 (12.9%)	404,973 (12.7%)	403,886 (12.3%)	411,715 (12.2%)	511,966 (12.3%)
Sex							
Female	1,426,569 (51.3%)	1,559,967 (51.2%)	1,602,926 (51.2%)	1,639,500 (51.3%)	1,682,566 (51.3%)	1,736,276 (51.3%)	2,141,081 (51.2%)
Male	1,355,492 (48.7%)	1,484,556 (48.8%)	1,526,670 (48.8%)	1,557,832 (48.7%)	1,598,463 (48.7%)	1,650,121 (48.7%)	2,038,708 (48.8%)
Duration of OHIP eligibility prior to calendar year							
<5 years	127,217 (4.6%)	128,117 (4.2%)	121,260 (3.9%)	118,520 (3.7%)	116,088 (3.5%)	117,061 (3.5%)	126,316 (3.0%)
59 years	168,176 (6.1%)	149,453 (4.9%)	153,498 (4.9%)	158,155 (5.0%)	159,779 (4.9%)	162,162 (4.8%)	158,973 (3.8%)
≥10 years	2,486,668 (89.4%)	2,766,953 (90.9%)	2,854,838 (91.2%)	2,920,657 (91.4%)	3,005,161 (91.6%)	3,107,175 (91.8%)	3,894,500 (93.2%)
Up to date status with colorectal evaluative procedures							
Overall percent of eligible population up to date	628,214 (22.6%)	960,998 (31.6%)	1,124,031(36.3%)	1,351,982 (42.4%)	1,614,445 (48.4%)	1,772,621(52.6%)	2,584,570 (62.2%)
Up to date by fecal occult blood testing	205,122 (7.4%)	347,530 (11.5%)	428,250 (13.7%)	537,957 (16.9%)	674,601 (20.7%)	715,528 (21.2%)	777,909 (18.7%)
Up to date by flexible sigmoidoscopy	28,926 (1.0%)	20,684 (0.7%)	17,988 (0.6%)	15,567 (0.5%)	13,656 (0.4%)	11,253 (0.3%)	5,778 (0.1%)
Up to date by CT colonography (from 2011 onward)	0	0	0	0	0	0	3,998 (0.1%)
Up to date by colonoscopy	394,167 (14.2%)	592,784 (19.5%)	687,793 (22.1%)	798,459 (25.1%)	926,189 (28.4%)	1,045,841 (31.0%)	1,796,886 (43.2%)

### Trends in Being Up-to-Date With Colorectal Evaluative Procedures in Ontario Among Screening-Eligible Persons

Between 2002 and 2007, screening-eligible persons up-to-date with any procedure increased from 628,214/2,782,061 (22.6%) to 1,351,982/3,197,332(42.4%). In 2008 (the year in which CCC was introduced), 1,614,445/3,281,028 (48.4%) screening eligibles were up-to-date, increasing to 2,584,570/4,179,789 (62.2%) in 2016. The absolute increase in the percent of colorectal screening eligibles up-to-date with any colorectal evaluative procedure from 2007 (the year prior to the introduction of CCC) to 2016 was 19.8% (from 1,351,982/3,197,332 to 2,584,570/4,179,789 persons).

Prior to the introduction of CCC, screening eligibles up-to-date with FOBT testing increased from 205,122/2,782,061 (7.4%) in 2002 to 537,957/3,197,332 (16.9%) in 2007. Although 674,601/3,281,028 (20.7%) were up-to-date with FOBT in the year CCC was introduced, by 2016 this was true for only 777,909/4,179,789 (18.7%). If a person had an FOBT followed by colonoscopy, the person was counted as up-to-date with FOBT for 24 months following the date of the FOBT, and then up-to-date with colonoscopy for 8 years (10 years minus the 24 months following the FOBT). Among the subpopulation of those not up-to-date with colonoscopy, those up-to-date with FOBT increased from 205,122/2,387,894 (8.6%) in 2002 to 674,601/2,354,839 (28.7%) in 2008 to 777,909/2,382,903 (32.7%) in 2016. By contrast, those up-to-date with colonoscopy increased from 394,167/2,782,061 (14.2%) to 798,459/3,197,332 (25.1%) in 2007 and from 926,189/3,281,028 (28.4%) to 1,796,886/4,179,789 (43.2%) after the introduction of CCC.

By 2016, 65% of females (1,378,541/2,131,064) and 60% of males (1,206,030/2,024,739) were up-to-date with colorectal evaluative procedures. By age group, 52% (576,725/1,107,504) of 51- to 54-year olds, 61% (614,174/1,000,534) 55- to 59-year olds, 66% (547,952/830,278) of 60- to 64-year olds, 70% (498,027/713,147) of 65- to 69-year olds and 69% (347,694/504,340) of 70- to 74-year olds were up-to-date. Annual tabulation of the percent up-to-date with colorectal evaluative procedures overall and stratified by procedure type is presented in Table 2.

**Table 2.** Annual screening-eligible population and percent up-to-date with colorectal evaluative procedures

Year	Screening-eligible population	Fecal occult blood test	Flexible sigmoidoscopy	CT colonography	Colonoscopy	Not up-to-date
2002	2,782,061	205,122 (7.4 %)	28,926 (1.0 %)		394,167 (14.2%)	2,153,847 (77.4%)
2003	2,864,326	244,065 (8.5 %)	26,399 (0.9 %)		450,396 (15.7%)	2,143,468 (74.8%)
2004	2,947,118	294,150 (10.0 %)	23,619 (0.8 %)		515,117 (17.5%)	2,114,233 (71.7%)
2005	3,036,504	347,530 (11.4 %)	20,684 (0.7 %)		592,784 (19.5%)	2,075,506 (68.4%)
2006	3,119,550	428,250 (13.7 %)	17,988 (0.6 %)		687,793 (22.0%)	1,985,519 (63.6%)
2007	3,185,388	537,957 (16.9 %)	15,567 (0.5 %)		798,459 (25.1%)	1,833,406 (57.6%)
2008	3,267,304	674,601 (20.6 %)	13,656 (0.4 %)		926,189 (28.3%)	1,652,859 (50.6%)
2009	3,370,896	715,528 (21.2 %)	11,253 (0.3 %)		1,045,841 (31.0%)	1,598,275 (47.4%)
2010	3,479,900	668,600 (19.2 %)	9,748 (0.3 %)		1,177,618 (33.8%)	1,623,934 (46.7%)
2011	3,593,697	714,525 (19.9 %)	8,561 (0.2 %)	383 (0.0 %)	1,302,615 (36.2%)	1,567,614 (43.6%)
2012	3,707,679	729,332 (19.7 %)	7,552 (0.2 %)	1,158 (0.0 %)	1,416,147 (38.2%)	1,553,491 (41.9%)
2013	3,825,809	713,071 (18.6 %)	6,983 (0.2 %)	2,011 (0.1 %)	1,527,372 (39.9%)	1,576,374 (41.2%)
2014	3,948,246	747,057 (18.9 %)	6,539 (0.2 %)	2,910 (0.1 %)	1,626,628 (41.2%)	1,565,113 (39.6%)
2015	4,058,706	768,730 (18.9 %)	6,083 (0.1 %)	3,744 (0.1 %)	1,719,218 (42.4%)	1,560,932 (38.5%)
2016	4,155,803	777,909 (18.7 %)	5,778 (0.1 %)	3,998 (0.1 %)	1,796,886 (43.2%)	1,571,233 (37.8%)

### Trends in CRC Incidence in Ontario Among Screening-Eligible Persons

Overall incidence decreased from 3,597 cases/2,782,061 (129.3 per 100,000, 95% CI 125.1, 133.6) in 2002, to 3,929 cases/4,155,803 (94.5 per 100,000, 95% CI 91.6, 97.6) in 2016 (two-sided Cochran–Armitage test for trend *P* < 0.0001). Among screening age-eligible males, CRC declined from 2,120 cases/1,355,492 or 156.4 per 100,000 (95% CI 149.9, 163.2) to 2,222 cases/2,038,708 or 114.9 per 100,000 (95% CI 110.3, 119.6) (two-sided Cochran–Armitage test for trend *P* < 0.0001), and among females, CRC declined from 1,477 cases/1,426,569 or 103.5 per 100,000 (95% CI 98.4, 109.0) to 1,600 cases/2,141,081 or 75.3 per 100,000 (95% CI 71.7, 79.0) (two-sided Cochran–Armitage test for trend *P* < 0.0001). In all age strata excepting age 51 to 54, there was a significant decline in incidence. Among those aged 51 to 54 years, overall incidence was 369 cases/810,730 or 45.5 per 100,000 (95% CI 41.1, 50.4) in 2002 and 517 cases/1,108,258 or 45.0 per 100,000 (95% CI 41.2, 49.1) in 2016, with a peak at 474 cases/939,021 or 50.5 per 100,000 (95% CI 46.2, 55.3) in 2008. Among the 5-year age strata between age 55 and 74 years, the decrease from 2002 to 2016 was steadily more profound: from 569 cases/637,926 or 89.2 per 100,000 (95% CI 82.2, 96.8) to 650 cases/1,003,582 or 68.1 per 100,000 (95% CI 63.1, 73.4) (*P* < 0.0001) among those aged 55 to 59 years, and from 1,032 cases/392,776 or 262.7 per 100,000 (95% CI 247.2, 279.3) to 897 cases/511,966 or 172.9 per 100,000 (95% CI 161.8, 184.8) (*P* < 0.0001) among those aged 70 to 74 years (Table 3).

**Table 3.** Annual incidence of first colorectal cancer (International Classification of Diseases codes C18, C19, plus C20) per 100,000 screening-eligible permanent residents, overall and stratified by sex and age

Year	Screening-eligible population (95% confidence interval)	Female (95% confidence interval)	Male (95% confidence interval)	Age 51–54 (95% confidence interval)	Age 55–59 (95% confidence interval)	Age 60–64 (95% confidence interval)	Age 65–69 (95% confidence interval)	Age 70–74 (95% confidence interval)
2002	129.3 (125.1, 133.6)	103.5 (98.4, 109)	156.4 (149.9, 163.2)	45.5 (41.1, 50.4)	89.2 (82.2, 96.8)	141.7 (131.7, 152.5)	208.8 (195.7, 222.8)	262.7 (247.2, 279.3)
2003	124.4 (120.4, 128.6)	101 (96, 106.3)	149.1 (142.8, 155.6)	45.3 (41, 50.2)	83.3 (76.7, 90.4)	142 (132.1, 152.6)	209.6 (196.6, 223.6)	241.4 (226.6, 257.2)
2004	127.8 (123.7, 131.9)	100.5 (95.5, 105.6)	156.5 (150.2, 163.1)	48.1 (43.6, 53)	85.8 (79.3, 92.9)	141.4 (131.7, 151.8)	209 (196.1, 222.8)	261.2 (245.8, 277.6)
2005	126.1 (122.1, 130.1)	100.7 (95.8, 105.8)	152.9 (146.7, 159.3)	52 (47.4, 57)	90.8 (84.2, 97.9)	135.7 (126.4, 145.7)	199.5 (187, 212.9)	255.2 (240, 271.4)
2006	122.2 (118.4, 126.1)	92.3 (87.7, 97.2)	153.7 (147.6, 160)	48.7 (44.4, 53.5)	84.2 (78, 90.8)	134.8 (125.7, 144.7)	209.1 (196.4, 222.6)	241.8 (227, 257.6)
2007	122.8 (119, 126.7)	97.7 (93, 102.6)	149.2 (143.3, 155.4)	50.2 (45.8, 55)	92.1 (85.6, 99.1)	137.5 (128.5, 147.1)	202.9 (190.5, 216.2)	231.9 (217.5, 247.3)
2008	125 (121.2, 128.9)	94.7 (90.1, 99.5)	157 (151, 163.3)	50.5 (46.2, 55.3)	88.7 (82.4, 95.5)	135 (126.3, 144.2)	204 (191.7, 217.1)	259.4 (244.1, 275.7)
2009	117.1 (113.6, 120.9)	95.7 (91.2, 100.4)	139.8 (134.2, 145.6)	49.5 (45.2, 54.1)	79.4 (73.5, 85.8)	130.7 (122.4, 139.6)	189.1 (177.5, 201.4)	240.8 (226.2, 256.3)
2010	111.9 (108.4, 115.5)	89 (84.8, 93.5)	136.1 (130.6, 141.7)	49.4 (45.2, 53.9)	82.7 (76.7, 89.1)	122.2 (114.4, 130.6)	178.7 (167.7, 190.5)	217.7 (204, 232.4)
2011	110.6 (107.2, 114.1)	87.4 (83.2, 91.8)	135.1 (129.7, 140.6)	49.8 (45.7, 54.3)	78.8 (73.1, 85)	124.3 (116.6, 132.6)	178.7 (167.8, 190.3)	210.7 (197.3, 225)
2012	105.9 (102.7, 109.3)	88.1 (84, 92.5)	124.7 (119.6, 129.9)	46.3 (42.4, 50.7)	74.9 (69.4, 80.8)	118.9 (111.3, 126.9)	169.8 (159.5, 180.7)	205.4 (192.3, 219.3)
2013	101.5 (98.3, 104.7)	81.7 (77.8, 85.8)	122.3 (117.3, 127.4)	43.7 (39.9, 47.9)	74.8 (69.4, 80.6)	109.5 (102.3, 117.2)	154.3 (144.8, 164.4)	206.5 (193.6, 220.1)
2014	99.8 (96.8, 103)	83.3 (79.4, 87.4)	117.3 (112.5, 122.2)	42.9 (39.2, 47)	73.5 (68.2, 79.1)	113.9 (106.7, 121.6)	143.2 (134.3, 152.7)	201.8 (189.4, 215.1)
2015	97.5 (94.5, 100.6)	77.6 (73.9, 81.5)	118.4 (113.7, 123.3)	49.3 (45.3, 53.6)	71.7 (66.6, 77.3)	98.9 (92.3, 106)	150 (141.1, 159.5)	182.6 (171, 194.9)
2016	94.5 (91.6, 97.5)	75.3 (71.7, 79)	114.9 (110.3, 119.6)	45 (41.2, 49.1)	68.1 (63.1, 73.4)	101.7 (95, 108.7)	145.3 (136.7, 154.4)	172.9 (161.8, 184.8)
Cochran–Armitage trend test (double sided)								
*P*	<0.0001	<0.0001	<0.0001	0.13	<0.0001	<0.0001	<0.0001	<0.0001

The incidence of colon cancer (ICD10 C18) declined significantly overall from 2,317 cases/2,782,061 or 83.3 per 100,000 (95% CI 80.0, 86.7) in 2002 to 2,554 cases/4,179,789 or 64.0 per 100,000 (95% CI 61.6, 66.5) (*P* < 0.0001) in 2016 among the screening age-eligible population. Among males, colon cancer declined from 1,276/1,355,492 or 94.1 per 100,000 (95% CI 89.1, 99.4) to 1,430/2,038,708 or 73.9 (95% CI 70.3, 77.8) (*P* < 0.0001), and among females, from 1,041/1,426,569 or 73.0 per 100,000 (95% CI 68.7, 77.5) to 1,124/2,141,081 or 54.6 per 100,000 (95% CI 51.5, 57.8) (*P* < 0.0001). Significant declines in colon cancer incidence were observed in the 5-year age strata between age 55 and 74 (all *P* < 0.0001).

The incidence of rectosigmoid plus rectal cancer (ICD10 C19 + C20) declined from 1,317 cases/2,782,061 or 47.3 per 100,000 (95% CI 44.9, 50.0) in 2002 to 1,350 cases/4,179,789 or 33.8 per 100,000 (95% CI 20.9, 24.9) in 2016 (*P* < 0.0001), and also significantly by sex. Among males rectosigmoid plus rectal cancer incidence declined from 867 cases/1,355,492 or 64.0 per 100,000 (95% CI 59.8, 68.4) to 844 cases/2,038,708 or 45.4 per 100,000 (95% CI 42.6, 48.4) among males (*P* < 0.0001); among females the corresponding decrease was from 451 cases/1,426,569 or 31.6 per 100,000 (95% CI 28.8, 34.7) to 506 cases/2,141,081 or 22.8 per 100,000 (95% CI 20.9, 24.9) (*P* < 0.0001). Among other age groups other than persons 51 to 54 years old, the incidence of rectosigmoid plus rectal cancer declined significantly (*P* = 0.004 to *P* < 0.0001).

### Trends in Incidence-Based CRC Mortality in Ontario in the Colorectal Screening-Age Group

Incidence-based CRC mortality declined from 1,141 CRC deaths/2,796,871 at risk for CRC death between ages 51 and 74 (40.8 per 100,000, 95% CI 38.5, 43.2) in 2002, to 1,009 CRC deaths/4,181,545 at risk (24.1 per 100,000, 95% CI 22.7, 25.7) in 2016 (*P* < 0.0001) (Table 4). Among males, incidence-based CRC mortality declined from 713 CRC deaths/1,364,099 at risk or 52.3 per 100,000 (95% CI 48.6, 56.2), to 601 CRC deaths/2,080,244 at risk, or 29.5 per 100,000 (95% CI 27.2, 31.9) (*P* < 0.0001), whereas among females, the decline was from 428 CRC deaths/1,432,772 at risk, or 29.9 per 100,000 (95% CI 27.2, 32.8) to 408 CRC deaths/2,186,116 at risk, or 19.0 per 100,000 (95% CI 17.3, 21.0) (*P* < 0.0001). Excepting the age stratum 51 to 54 years, decreases in incidence-based CRC mortality in all other age strata were statistically significant by Cochran–Armitage tests for trend (*P* < 0.0001) (Table 4).

**Table 4.** Annual incidence-based colorectal cancer mortality per 100,000 screening age population

Year	Screening-age population (95% confidence interval)	Female (95% confidence interval)	Male (95% confidence interval)	Age 51–54 (95% confidence interval)	Age 55–59 (95% confidence interval)	Age 60–64 (95% confidence interval)	Age 65–69 (95% confidence interval)	Age 70–74 (95% confidence interval)
2002	40.8 (38.5, 43.2)	29.9 (27.2, 32.8)	52.3 (48.6, 56.2)	7.0 (5.4, 9.1)	24.5 (21.0, 28.7)	47.0 (41.4, 53.4)	75.7 (68.0, 84.3)	89.2 (80.4, 99.0)
2003	37.9 (35.7, 40.2)	28.6 (26.0, 31.5)	47.6 (44.1, 51.4)	7.8 (6.1, 10.0)	23.3 (20.0, 27.2)	42.0 (36.8, 47.9)	65.8 (58.7, 73.8)	88.0 (79.3, 97.7)
2004	37.2 (35.1, 39.5)	28.7 (26.1, 31.5)	46.2 (42.8, 49.8)	6.7 (5.1, 8.7)	22.7 (19.5, 26.5)	43.2 (38.0, 49.1)	61.8 (55.0, 69.5)	91.1 (82.3, 100.9)
2005	35.1 (33.0, 37.2)	25.1 (22.8, 27.7)	45.5 (42.2, 49.1)	8.3 (6.6, 10.5)	22.0 (18.9, 25.6)	34.2 (29.7, 39.4)	62.7 (55.9, 70.3)	86.3 (77.7, 95.8)
2006	32.5 (30.5, 34.5)	24.1 (21.8, 26.6)	41.3 (38.2, 44.7)	7.8 (6.2, 9.9)	18.8 (16.0, 22.1)	37.6 (32.9, 42.9)	57.1 (50.6, 64.3)	77.7 (69.6, 86.8)
2007	32.0 (30.1, 34.1)	24.3 (22.0, 26.8)	40.2 (37.2, 43.5)	8.6 (6.9, 10.8)	20.1 (17.2, 23.5)	35.3 (30.9, 40.3)	57.1 (50.7, 64.3)	73.5 (65.7, 82.3)
2008	31.5 (29.7, 33.5)	22.5 (20.4, 24.9)	41.0 (38.0, 44.3)	8.1 (6.5, 10.1)	18.5 (15.8, 21.8)	34.6 (30.4, 39.4)	55.5 (49.3, 62.5)	77.2 (69.1, 86.2)
2009	29.5 (27.7, 31.4)	22.0 (19.9, 24.3)	37.4 (34.5, 40.4)	6.4 (5.0, 8.2)	21.1 (18.2, 24.5)	31.9 (27.9, 36.4)	45.1 (39.6, 51.3)	77.0 (69.0, 85.9)
2010	25.7 (24.1, 27.5)	18.6 (16.7, 20.7)	33.2 (30.6, 36.1)	4.8 (3.6, 6.4)	16.5 (14.0, 19.5)	30.0 (26.3, 34.3)	46.4 (41.0, 52.6)	59.9 (53.0, 67.8)
2011	28.1 (26.4, 29.9)	21.3 (19.3, 23.5)	35.2 (32.6, 38.1)	6.8 (5.4, 8.6)	18.3 (15.7, 21.4)	30.6 (26.9, 34.9)	49.3 (43.8, 55.6)	66.8 (59.5, 74.9)
2012	26.6 (25.0, 28.3)	19.7 (17.8, 21.8)	33.9 (31.3, 36.7)	7.4 (6.0, 9.3)	17.5 (14.9, 20.4)	31.4 (27.7, 35.7)	41.4 (36.6, 47.0)	62.9 (55.9, 70.7)
2013	24.3 (22.8, 25.9)	19.6 (17.8, 21.7)	29.2 (26.8, 31.7)	4.8 (3.6, 6.3)	12.8 (10.7, 15.3)	30.6 (26.9, 34.8)	42.0 (37.3, 47.4)	58.4 (51.8, 65.8)
2014	25.2 (23.7, 26.8)	19.9 (18.0, 21.9)	30.8 (28.4, 33.3)	5.1 (3.9, 6.6)	14.8 (12.6, 17.5)	28.6 (25.1, 32.6)	42.7 (38.0, 47.9)	62.1 (55.4, 69.6)
2015	24.8 (23.3, 26.4)	19.9 (18.1, 21.9)	30.0 (27.7, 32.5)	7.2 (5.8, 8.9)	16.1 (13.8, 18.8)	26.5 (23.1, 30.2)	42.7 (38.1, 47.9)	53.6 (47.5, 60.4)
2016	24.1 (22.7, 25.7)	19.0 (17.3, 21.0)	29.5 (27.2, 31.9)	4.9 (3.7, 6.4)	17.7 (15.3, 20.5)	27.4 (24.1, 31.2)	42.2 (37.7, 47.2)	47.7 (42.1, 54.1)
Cochran– Armitage trend test	*P* < 0.0001	*P* < 0.0001	*P* < 0.0001	*P* = 0.127	*P* < 0.0001	*P* < 0.0001	*P* < 0.0001	*P* < 0.0001

## Discussion

Following the introduction of CCC in 2008, the percent of screening eligibles up-to-date with colonoscopy continued to increase, whereas those up-to-date with FOBT did not increase. Among those not up-to-date with colonoscopy, there was a modest increase in the percent up-to-date with FOBT from 674,601/2,354,839 (28.7%) in 2008 to 777,909/2,382,903 (32.7%) in 2016.

Several factors underlie the lack of increase in the percent up-to-date with FOBT. Although there is high-quality evidence that mailing FOBT kits to screening eligibles increases participation in screening (11), this was not a component of CCC. In addition, primary care practitioners in Ontario believe that FOBT is inferior to colonoscopy as a screening test, and therefore often do not recommend it to their patients (12). In 2016, the percent up-to-date with colorectal evaluative procedures 8 years after the introduction of CCC was similar to the level of self-reported colorectal screening status in the 2015 United States National Health Interview Survey, which found that recent colorectal screening was reported by 63.4% of age-eligible females (compared to 65% observed to be up-to-date in Ontario) and 61.9% of age-eligible males (compared to 60% observed to be up-to-date in Ontario) (13). While increases in colorectal screening participation in North America are encouraging, further improvements in the prevention and early detection of CRC are required.

An international collaborative group of cancer screening researchers has examined barriers to effective screening, especially factors within screening programs as well as external health system factors, using the Barriers to Effective Screening Tool (BEST) (14,15). BEST was applied among cancer screening organizations, screening researchers and health policymakers, as well as by systematic literature review (16). Many of the barriers are outside the direct control of cancer screening programs, for example, incomplete and/or inaccurate lists of eligibles and their addresses (15), difficulties in access to screening (17), insufficient coordination (14,15,17) and expenditures on opportunistic screening (14,16). Some expenditures on opportunistic screening may add little to the effectiveness of colorectal screening in Ontario; however, if those resources were to be reallocated to programmatic screening, the levels of participation and effectiveness of screening might be enhanced. We have previously shown that 33.7% of patients in the colorectal screening-eligible age range who underwent a complete negative outpatient colonoscopy, without prior, new or subsequent diagnoses of CRC or inflammatory bowel disease, went on to have a repeat colonoscopy between 0.5 and 5.5 years later (18). During that follow-up window, only 0.5% received a new diagnosis of CRC or inflammatory bowel disease, or underwent bowel resection for any reason in the interval (18).

We have found statistically significant decreases in CRC incidence between 2002 and 2016, overall, and stratified by anatomic site, sex and age, continuing previously observed downward secular trends in CRC incidence in Canada (1983 to 2007) (1) and the United States (19,20). These decreases began earlier than widespread screening and colonoscopy utilization, and so should not be ascribed entirely to the impact of widespread colorectal screening on CRC prevention. Nevertheless, as in the United States (19), much of the decline in CRC incidence in Ontario is likely due to the increasing prevalence of colonoscopy for screening and other indications. We did not find a decrease in CRC incidence among 51- to 54-year-old persons. The incidence of rectosigmoid plus rectal carcinoma among this age group was higher in 2016 than in 2002. Colonoscopic polypectomy performed during the first few years of screening age-eligibility would not be expected to decrease incidence of CRC among those aged 51 to 54.

We have found statistically significant decreases in incidence-based CRC mortality 2002 to 2016, similar to long-term declines in CRC mortality documented in the Canada (1), United States (21) and Europe (22), which occurred in advance of large increases in the uptake of colorectal evaluative procedures and screening and in advance of the introduction of organized colorectal screening programs. The effectiveness of colonoscopic polypectomy in preventing CRC likely results in decreased CRC mortality more than a decade after the procedure (2,3). The decline in CRC incidence associated with the increasing prevalence of colonoscopic polypectomy provided in Ontario since the decade of the 1990s (7) is the most likely explanation for much of the decline in incidence-based CRC mortality across the entire time period of observation to 2016 (23). It is possible that some of the decline in incidence-based CRC mortality could be attributable the prior secular trends in CRC incidence and mortality (1), and also to better access to appropriate surgery and chemotherapy for CRC, both of which have been given a high priority by Cancer Care Ontario.

The strengths of this study include the availability of all records of all colorectal evaluative procedures, CRC diagnoses and deaths from CRC, which are of good quality (24,25). The entire geographically defined target population itself is a major strength, whose number increased from 2,782,061 to 4,179,789 persons between 2002 and 2016, including marginalized subpopulations and those who experience barriers to care. Over 90% of screening-eligible persons had residence in Ontario for >10 years, so the possibility of misclassification of any person’s history of colorectal evaluative procedures or CRC is low. Limitations include the absence of information on the indication for colorectal evaluative procedures throughout the study period, and the inability to examine trends in FOBT positivity before and after the introduction of CCC. It is unknown if the fecal immunochemical test would have been associated with a higher percentage of persons up-to-date with stool testing. We emphasize that the decreases in incidence and incidence-based mortality are confined to the typical screening age-eligible population, that we did not observe decreases in the 51- to 54-year-old age group, and that our findings must not be extrapolated to those younger or older than the typical screening age-eligible population.

## Conclusion

The percent of screening age-eligible permanent residents of Ontario up-to-date with colonoscopy continued to increase annually during 2002 to 2016, following the previously observed increased utilization in 1992 to 2001, which was likely the most significant factor in the decline of CRC incidence and incidence-based mortality in Ontario from 2002 to 2016, and likely more important than other factors such as long-term gradual declines in incidence and mortality, or the impact on mortality of improved treatment.

## Funding

This work was supported by a grant from Cancer Care Ontario to LFP. This study was also supported by ICES, which is funded by an annual grant from the Ontario Ministry of Health and Long-Term Care (MOHLTC). The funder had no role in the design of the study or collection, analysis and interpretation of the data or in writing the manuscript. The opinions, results, views and conclusions reported in this paper are those of the authors and do not necessarily reflect those of the ICES, the MOHLTC or Cancer Care Ontario, and no endorsement by these bodies is intended or should be inferred.
